# Defining aggressive prostate cancer: a geospatial perspective

**DOI:** 10.1186/s12885-023-11281-8

**Published:** 2023-08-14

**Authors:** Daniel Wiese, Tesla D. DuBois, Kristen A. Sorice, Carolyn Y. Fang, Camille Ragin, Mary B. Daly, Adam C. Reese, Kevin A. Henry, Shannon M. Lynch

**Affiliations:** 1 Department of Surveillance and Health Equity Science, American Cancer Society, 3380 Chastain Meadows Pkwy NW Suite 200, Kennesaw, GA 30144 USA; 2 Department of Geography, Temple University, Philadelphia, PA USA; 3 Cancer Prevention and Control, Fox Chase Cancer Center, Philadelphia, PA USA; 4 Temple Health Urology, Philadelphia, PA USA

**Keywords:** Prostate cancer, Geospatial, Aggressive prostate cancer, Survival analysis

## Abstract

**Background:**

Spatial analysis can identify communities where men are at risk for aggressive prostate cancer (PCan) and need intervention. However, there are several definitions for aggressive PCan. In this study, we evaluate geospatial patterns of 3 different aggressive PCan definitions in relation to PCan-specific mortality and provide methodologic and practical insights into how each definition may affect intervention targets.

**Methods:**

Using the Pennsylvania State Cancer Registry data (2005–2015), we used 3 definitions to assign “aggressive” status to patients diagnosed with PCan. Definition one (D1, recently recommended as the primary definition, given high correlation with PCan death) was based on staging criteria T4/N1/M1 or Gleason score ≥ 8. Definition two (D2, most frequently-used definition in geospatial studies) included distant SEER summary stage. Definition three (D3) included Gleason score ≥ 7 only. Using Bayesian spatial models, we identified geographic clusters of elevated odds ratios for aggressive PCan (binomial model) for each definition and compared overlap between those clusters to clusters of elevated hazard ratios for PCan-specific mortality (Cox regression).

**Results:**

The number of “aggressive” PCan cases varied by definition, and influenced quantity, location, and extent/size of geographic clusters in binomial models. While spatial patterns overlapped across all three definitions, using D2 in binomial models provided results most akin to PCan-specific mortality clusters as identified through Cox regression. This approach resulted in fewer clusters for targeted intervention and less sensitive to missing data compared to definitions that rely on clinical TNM staging.

**Conclusions:**

Using D2, based on distant SEER summary stage, in future research may facilitate consistency and allow for standardized comparison across geospatial studies.

**Supplementary Information:**

The online version contains supplementary material available at 10.1186/s12885-023-11281-8.

## Introduction

Prostate cancer (PCan) has the highest incidence rate of all cancers diagnosed in men in the United States [1]. While incidence rates have dropped since the early 1990s [2], PCan remains a large burden nationally, resulting in an estimated 34.5 thousand deaths in 2022 [1]. PCan burden is also high in Pennsylvania, where approximately 11,740 PCan cases will be diagnosed and 1,470 PCan deaths will occur in 2022 [1]. It is estimated that 1 in 9 men in Pennsylvania will develop PCan in their lifetime [3].

Although the overall 5-year survival rate of 95% for PCan is relatively high, there are substantial differences by stage. In contrast to the local and regional stages, both of which have a 5-year survival rate of nearly 100%, only 31% of all patients survive five years after being diagnosed with distant stage [1]. This is a major public health concern, as rates of distant stage diagnoses have increased substantially since 2010 [4]. The key to reducing PCan deaths is a diagnosis at an early stage and receipt of proper treatment [5]. However, the benefits of PCan screening may be questionable, as years of early prostate-specific antigen (PSA) screening may have resulted in over-diagnosis and unnecessary treatment of PCan, especially among men under age 50 [5, 6].

Defining the aggressive form of PCan more precisely would reduce unnecessary treatment and decrease PCan mortality. However, doing so is challenging due to differences in potential etiologic risk factors between indolent and aggressive forms of PCan [7]. Several attempts have been made to define the aggressive form of PCan in genetic and environmental risk factor studies. In these studies, stage (localized, regional, distant or TNM) and/or Gleason score (≥ 7 or ≥ 8) of PCan have been used to determine disease aggressiveness [8–10]. However, guidelines used by epidemiologists, clinicians, and pathologists continue to vary.

To address this gap, a recent study by Hurwitz and colleagues [6] evaluated several definitions of aggressive PCan as they relate to deaths in prospective cohort and registry studies. They found that the most aggressive form should be defined as a combination of stage (T4 or N1 or M1) or grade (Gleason score ≥ 8) because it was most effective in a sensitivity analysis among 12 definitions in correlating with PCan deaths. However, while the authors argue that TNM is a widely used classification schema, there are several issues related to its accuracy and availability in cancer registry records. In contrast to the registry-derived codes (e.g., SEER summary stage), which have been used in their original form for several decades [8], the TNM classification is more dynamic. Employed primarily by clinicians [9], the TNM classification is derived from the AJCC recommendations, which have had several updates since their initial implementation. Concerns around delays in code adaptation [10] and inconsistency in the staging between different AJCC versions [11, 12] are well-documented.

Population-based registry data is often used for geospatial analysis studies, which could aid intervention planning [13]. However, various definitions of aggressive PCan would influence geospatial modeling [14] and may impact the planning of cancer-related interventions. The objective of our study was to evaluate the recently proposed definition of aggressive PCan (T4 or N1 or M1 or Gleason score ≥ 8) in contrast to two alternatives (SEER summary distance stage and Gleason score ≥ 7) in a geospatial context. Our analysis included all Pennsylvania patients diagnosed with PCan between 2005 and 2015 and followed through the end of 2017. Using that subset, we built a Bayesian spatial model to estimate (1) statistically significant clusters of elevated odds ratios using each definition of the aggressive PCan, and (2) the geographic risk of death (e.g., spatial hazard ratio) from PCan. We then compared the geographic location of statistically significant clusters of elevated aggressive PCan odds ratios and PCan-specific mortality. Finally, we summarized the socio-demographic factors of the clusters based on the population and patients’ characteristics.

## Methods

### Study population

Prostate cancer cases were obtained from the Pennsylvania State Cancer Registry (PCR). They included all Pennsylvania residents with a histologically confirmed first primary PCan, diagnosed between January 1, 2005, and December 31, 2015, according to the International Classification of Diseases for Oncology, 3rd Edition (ICD-03), and were followed until December 31, 2017. The selected time frame represents the last decade of continuous PCan incidence rate decline. There was a total of 97,608 PCan cases in the original database. Several exclusions (n = 15,028) were applied to preserve only cases with available staging information across all classification schemas: SEER summary stage, TNM, and Gleason score. However, demographic characteristics of the excluded cases did not substantially vary from the overall study population (results not shown). The final study population was 82,580 cases. Individual-level data included age and stage at diagnosis (SEER summary, TNM, Gleason Score), race (White, Black, Native American, Asian), date of last contact, vital status (dead/alive), cause of death, and patient’s primary address. All addresses were geocoded to the 2010 census tract level using ArcGIS 10.7 software [15]. The study was approved by Fox Chase Cancer Center’s institutional review board (IRB No. 18-9015).

### Definitions of aggressive prostate cancer

We developed three definitions of aggressive PCan for every patient record. The first definition was recently proposed by Hurwitz and colleagues [6]and is defined as T4 or N1 or M1 or Gleason score ≥ 8 (referenced as D1). The second definition included only distant stage cases as defined by the SEER summary stage (D2). The third definition was based on the clinical description using Gleason score ≥ 7 (D3). For purposes of this study, PCan cases were then grouped into two categories defined as ‘non-aggressive’ or ‘aggressive’ based on each of the three definitions.

### Statistical analysis

#### Estimating geographic odds ratios of elevated risk for aggressive prostate cancer

To investigate potential geographic variation in aggressive PCan compared to the non-aggressive form, we applied binomial Bayesian spatial models. The objective of this statistic is to detect census tracts with statistically significantly elevated odds ratios (OR) compared to the statewide average (e.g., geographic clustering). For each definition, we developed a separate model (three in total). All models were adjusted for the diagnosis year and the patient’s age at diagnosis. No adjustments for the patient’s race were applied to avoid eliminating any clusters that could be explained due to racial disparities because the purpose of this study is to detect geographic clusters of elevated ORs of aggressive PCan prior to looking at explanatory factors.

#### Estimating geographic risk of death from prostate cancer

For the geographic PCan-specific survival analysis, we calculated patient survival time in months as the difference between the date of diagnosis and the date-of-last-contact or death. Cases were censored at the date-of-last-contact or the end of the follow-up period (December 31, 2017), whichever occurred first. Cases missing vital status and follow-up information were excluded. The final study population included 79,031 patients. To estimate the geographic risk of death (e.g., Hazard Ratios = HR) for each census tract, we applied the Bayesian geoadditive model, which extends the conventional Cox regression survival model [16]. The model was adjusted for the age at diagnosis.

Both the binomial and the proportional hazards Cox regression models include a spatial function to estimate the spatial effect based on the geographic location of the patient’s census tract at the time of diagnosis after controlling for individual-level covariates [17, 18]. The regression models are based on Markov Chain Monte Carlo simulation techniques, corresponding to full Bayesian inference, and obtained by specifying prior distributions for all unknown parameters. For each model, we ran 10,000 iterations, with the first 2,000 samples used as a burn-in. The posterior distribution for each parameter estimate was constructed using every 20th sample from the remaining 8,000 samples. The 95% confidence intervals (CI) were used to designate significantly higher or lower estimates than the state average based on the posterior distribution of the 1,000 final samples [17]. All tests of statistical significance were 2-sided. The ORs and HRs are the exponentiated smoothed posterior mean for each census tract based on all patients residing there.

All models were applied using R packages R2BayesX [19] and BayesX [20]. The exponentiated spatial effects of each census tract were summarized for each cluster, and all statistically significant clusters of elevated ORs and HRs were mapped using QGIS v.3.10 [21].

### Comparing geographic odds of aggressive prostate cancer to risk of death from prostate cancer

Geographic clusters of elevated odds ratios from each model and PCan-specific hazard ratios were compared by summarizing socio-demographic characteristics and by visual interpretation. This approach was complemented with statistical calculations of sensitivity/specificity of each definition in accurately defining risk of PCan death (Supplementary Table 4).

## Results

### Study population

**Fig. 1:**
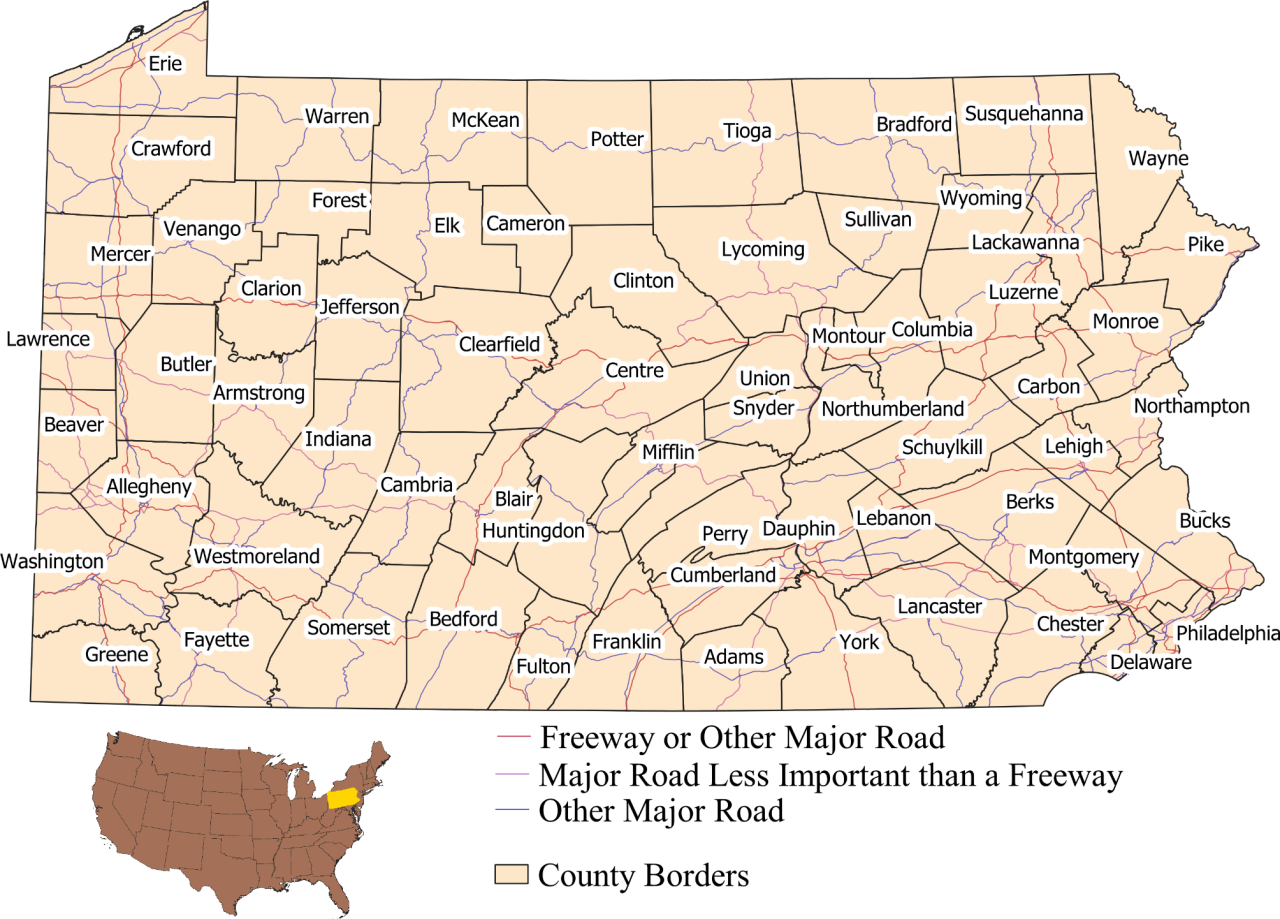
Pennsylvania county map. The study area included the entire Commonwealth of Pennsylvania, located in the Northeastern United States. Tan polygons with a black outline represent county boundaries

The study area included the state of Pennsylvania, located in the Northeastern United States (Fig. 1). The overall study population included 82,580 cases comprised of 88% White, and 11% Black patients. The average age at diagnosis was 66 years. The number of aggressive PCan cases varied by definition. According to D1, 19.2% (n = 15,818) were diagnosed with aggressive PCan, while only 4.2% (n = 3,474) of cases were considered aggressive using D2. Using D3 resulted in the largest quantity aggressive PCan cases, accounting for 29.5% of the sample (n = 24,354) (Table 1).

**Table 1:** Study population characteristics

Characteristics	Aggressiveness Analysis	Survival Analysis*
	n = 82,580 (%)	n = 79,031 (%)
Age		
Mean (Min, Max)	65.8 (31.0, 105)	65.8 (31.0, 105)
Race		
Asian	597 (0.7%)	569 (0.7%)
Black	9,427 (11.4%)	9,067 (11.4%)
Native American	33 (0.1%)	32 (0.1%)
White	72,523 (87.8%)	69,363 (87.8%)
Vital Status		
Censored	78,422 (95.0%)	74,892 (94.8%)
Prostate Cancer Death	4,158 (5.0%)	4,139 (5.2%)
Hurwitz et al Definition (D1)		
Non-Aggressive	66,762 (80.8%)	63,540 (80.4%)
Aggressive	15,818 (19.2%)	15,491 (19.6%)
SEER Distant Stage (D2)		
Non-Aggressive	79,106 (95.8%)	75,610 (95.7%)
Aggressive	3,474 (4.2%)	3,421 (4.3%)
High Gleason score ≥ 7 (D3)		
Non-Aggressive	58,226 (70.5%)	55,284 (70.0%)
Aggressive	24,354 (29.5%)	23,747 (30.0%)

Note: *3,549 (4.2%) cases excluded were due to missing or negative survival time

Analyzing the distribution by race and age for each definition, we found that using D1, Black patients accounted for 12% of aggressive cases, White patients accounted for 87%, and the average age of diagnosis was 70 years. Approximately 20% died from PCan (Table 2, left). Using D2, aggressive cases were comprised of 15% Black and 84% White patients, with an average age at diagnosis of 71 years. Approximately 55% of these patients died from PCan (Table 2, middle). Considering D3, 13% of the patients were Black, and 87% White. The average age at the diagnosis was 68 years. Approximately 14% of the patients in this definition died from PCan (Table 2, right). A comparison of characteristics between aggressive and non-aggressive PCan cases is provided in the supplementary file (Supplementary Tables 1–3).

**Table 2:** Study population of aggressive prostate cancer cases based on D1 (left), D2 (center), and D3 (right)

	D1*	D2**	D3***
Characteristics	n = 15,818 (%)	n = 3,474 (%)	n = 24,354 (%)
Age			
Mean (Min, Max)	69.4 (35.0, 99.0)	70.6 (38.0, 98.0)	68.4 (35.0, 99.0)
Race			
Asian	131 (0.8%)	28 (0.8%)	213 (0.9%)
Black	1,957 (12.4%)	519 (14.9%)	3,062 (12.6%)
Native American	6 (0.0%)	2 (0.1%)	7 (0.0%)
White	13,724 (86.8%)	2,925 (84.2%)	21,072 (86.5%)
Vital Status			
Censored	12,603 (79.7%)	1551 (44.6%)	21,047 (86.4%)
Prostate Cancer Death	3215 (20.3%)	1923 (55.4%)	3307 (13.6%)

* Definition one (D1) was based on staging criteria(T4, N1, M1) or Gleason score of ≥ 8

** Definition two (D2) was based on distant SEER summary stage

*** Definition three (D3) was based on the clinical Gleason score of ≥ 7 only

For survival analysis, the study population included 79,031 cases with available follow-up time and vital status. The average follow-up time was 22.7 months (median follow-up of 8 months). Of the 4,158 deaths, 13.5% were of Black patients, and 86% were White (Table 3). Among PCan deaths, 77% would be defined as aggressive, according to D1. A similar amount (79.5%) would be considered aggressive using D3. In contrast, the proportion of aggressive PCan cases among deaths would be only 46.2% based on definition D2 (Table 3).

**Table 3:** Study population characteristics of prostate cancer-specific deaths

	Death Events
Characteristics	n = 4,158 (%)
Age	
Mean (SD)	70.9 (10.7)
Median [Min, Max]	71.0 [35.0, 99.0]
Race	
Asian	21 (0.6%)
Black	563 (13.5%)
Native American	1 (0.0%)
White	3,573 (85.9%)
Hurwitz Definition D1	
Non-Aggressive	943 (22.7%)
Aggressive	3,215 (77.3%)
SEER Distant Stage D2	
Non-Aggressive	2,235 (53.8%)
Aggressive	1,923 (46.2%)
High Gleason 8 + D3	
Non-Aggressive	851 (20.5%)
Aggressive	3,307 (79.5%)

### Significant clusters of elevated odds ratios of aggressive prostate cancer

**Fig. 2:**
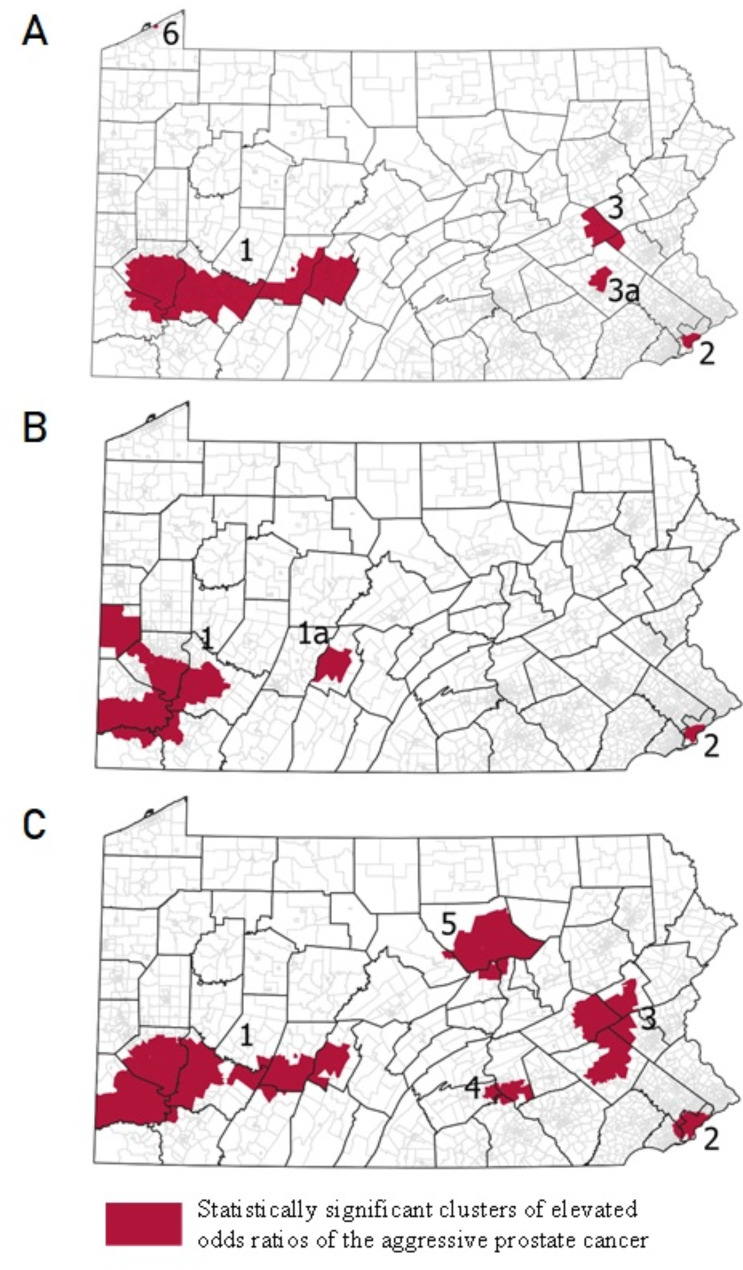
Location of statistically significant clusters (dark areas) of elevated odds ratios of the aggressive prostate cancer by definition. (Definitions: D1 = T4 or N1 or M1 or Gleason ≥ 8 (**A**); D2 = Distant SEER summary stage (**B**); D3 = Gleason ≥ 7 (**C**). Black lines represent county boundaries. Gray lines represent census tract boundaries

Analyzing the spatial modeling results from each definition, we found that while the number of statistically significant clusters of elevated ORs of aggressive PCan (numbered 1–6) varied, the locations were not dissimilar across the three definitions (Fig. 2A-C; Table 4).

**Table 4:** Cluster characteristics based on age-adjusted logistic regression models

Cases	Cluster 1	Cluster 1a	Cluster 2	Cluster 3	Cluster 3a	Cluster 4	Cluster 5	Cluster 6	Outside	
Counties	Allegheny to Blair	Blair Only	Philly	Schuylkill	Reading Only	Harrisburg	Lycoming	Erie		
Census Tracts (n)	497		137	7	31			1	2529	Definition = D1
Odds Ratio (CI)	1.35 (1.15–1.55)		1.10 (1.07–1.13)	1.22 (1.19–1.25)	1.14 (1.11–1.17)			1.16 (1.16–1.16)	0.97 (0.77–1.16)	
Cases										
Asian (%)	42 (0.4)		55 (2.17)		2 (0.31)				498 (0.73)	
Black (%)	1125 (10.66)		1270 (50.12)		82 (12.85)				6950 (10.12)	
Native (%)	2 (0.02)		2 (0.08)						29 (0.04)	
White (%)	9388 (88.93)		1207 (47.63)	148 (100)	554 (86.83)			30 (100)	61,196 (89.11)	
Aggressive (%)	2607 (24.69)		508 (20.05)	41 (27.7)	153 (23.98)			4 (13.3)	12,505 (18.21)	
Death (%)	654 (6.19)		169 (6.67)	17 (11.49)	27 (4.23)			1 (3.3)	3290 (4.79)	
Census Tracts (N)	447	24	178						2553	Definition = D2
Odds Ratio (CI)	1.32 (1.12–1.52)	1.45 (1.39–1.52)	1.23 (1.15–1.30)						0.95(0.63–1.28)	
Cases										
Asian (%)	22 (0.26)		65 (1.76)						510 (0.73)	
Black (%)	1136 (13.3)	13 (2.66)	2136 (57.68)						6142 (8.79)	
Native American (%)	1 (0.01)	1 (0.2)	3 (0.08)						28 (0.04)	
White (%)	7380 (86.43)	475 (97.14)	1499 (40.48)						63,169 (90.44)	
Aggressive (%)	535 (6.27)	45 (9.2)	208 (5.62)						2686 (3.85)	
Death (%)	575 (6.73)	52 (10.63)	251 (6.78)						3280 (4.7)	
Census Tracts (N)	551		386	83		64	29		2089	Definition = D3
Odds Ratio (CI)	1.4 (1.11–1.70)		1.14 (1.07–1.21)	1.18 (1.09–1.26)		1.15 (1.13–1.17)	1.47 (1.28–1.67)		0.98 (0.79–1.16)	
Cases										
Asian (%)	43 (0.36)		168 (1.86)	6 (0.28)		9 (0.87)	3 (0.33)		368 (0.64)	
Black (%)	1194 (9.94)		5000 (55.25)	118 (5.51)		195 (18.77)	33 (3.58)		2887 (5.03)	
Native (%)	3 (0.03)		5 (0.06)	1 (0.05)					24 (0.04)	
White (%)	10,772 (89.68)		3876 (42.83)	2016 (94.16)		835 (80.37)	885 (96.09)		54,139 (94.29)	
Aggressive (%)	4294 (35.75)		2748 (30.37)	736 (34.38)		321 (30.9)	353 (38.33)		15,902 (27.7)	
Death (%)	746 (6.21)		516 (5.7)	106 (4.95)		44 (4.23)	51 (5.54)		2695 (4.69)	

Note: CI = 95% Confidence Interval

Cluster 1, located in western Pennsylvania and including much of Allegheny County, was identified using all three definitions (Fig. 2A-C). While there are differences in the extent of Cluster 1 based on each model, models D1 (Fig. 2A- OR = 1.35; 95%CI = 1.15–1.55) and D3 (Fig. 2C-OR = 1.4; 95%CI = 1.11–1.70) are most similar, extending east of Allegheny County and to Blair County. By contrast, the model using D2 (Fig. 2B) resulted in two sub-clusters, one of which included Allegheny County and counties west of it (Cluster 1-OR = 1.32; 95%CI = 1.12–1.52) and a separate smaller Cluster 1a in Blair County (OR = 1.45; 95%CI = 1.39–1.52). Cluster 2 consistently remained in the Philadelphia area in all models, with only minor differences in the extent and OR estimates (OR = 1.10; 95%CI = 1.07–1.13 using D1; OR = 1.23; 95%CI = 1.15–1.30 using D2; OR = 1.14; 95%CI = 1.07–1.21 using D3). In contrast to model D2, models D1 and D3 identified additional clusters in the Lehigh Valley (Cluster 3) and Reading area (Cluster 3a). Moreover, the model using the definition D1 found another one-census-tract cluster in Erie County (Fig. 2A-Cluster 6). Two additional clusters in the Harrisburg area (Cluster 4) and Schuylkill County (Cluster 5) were detected using definition D3 (Fig. 2C) (Table 3). Comparing each cluster from every model, we found that even in consistently overlapping clusters (Clusters 1 and 2), ORs were highest when using definition D2 compared to D1 and D3 (Table 4).

### Significant clusters of elevated risk of death

**Fig. 3:**
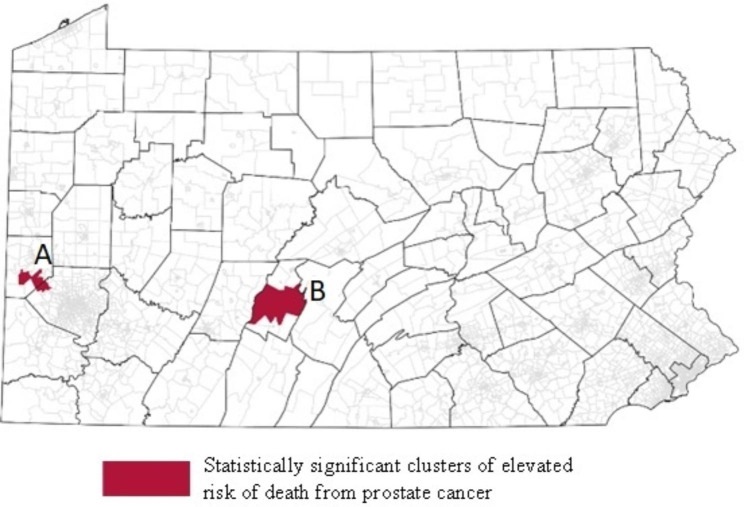
Location of significant clusters of elevated risk of death (dark areas) from prostate cancer. Black lines represent county boundaries. Gray lines represent census tract boundaries

The modeling results from the spatial PCan-specific survival analysis indicated only two statistically significant clusters of elevated risk of death (marked A and B), both in close proximity to each other (Fig. 3). The HR for Cluster A was 1.25 (95%CI = 1.21–1.29), and for Cluster B, it was 1.40 (95%CI = 1.34–1.46), indicating a 25% and 40% higher risk of death, respectively, compared to the statewide average.

### Comparison of significant geographic clusters of aggressive prostate cancer and elevated risk of death

Spatially, both clusters of elevated risk of death (Fig. 3) partially overlap with the clusters of aggressive prostate cancer detected in binomial models for each definition of aggressive PCan (Fig. 2A-C). While the risk of death Cluster A in Fig. 3 (Allegheny and Beaver Counties) overlaps partially with Cluster 1 from Fig. 2B of elevated ORs of aggressive PCan based on D2, Cluster B in Fig. 3 (Blair County) almost entirely overlaps with the areas found to have significantly higher ORs of aggressive PCan using any definition of aggressive prostate cancer (Fig. 2A-C). However, upon visual inspection, the D2 definition (Fig. 2B) most closely resembles the risk of death cluster map (Fig. 3). This is in line with sensitivity and specificity analyses conducted (Supplementary Table 4), which found D2 to have the highest degree of specificity (98%) with PCan death. However, we found considerable variation when comparing the number of aggressive PCan cases in each cluster based on the three definitions (Cluster A: D1 = 68, D2 = 17, D3 = 91; Cluster B: D1 = 151, D2 = 45, D3 = 182) (Table 5). This resulted in D3 reporting the highest sensitivity (79.5%; Supplementary Table 4).

**Table 5:** Patient characteristics within each cluster based on age-adjusted Cox proportional hazard regression model

Cases	Cluster A	Cluster B	Outside
Counties	Beaver, Allegheny	Blair	
Census tracts (n)	15	25	3177
Hazard Ratio	1.25 (1.21–1.29)	1.4 (1.34–1.46)	0.97 (0.70–1.24)
Cases			
Asian (%)			597 (0.73)
Black (%)	13 (4.33)	13 (2.56)	9401(11.5)
Native American (%)		1 (0.19)	32 (0.04)
White (%)	287 (95.67)	494 (97.24)	71,742 (87.73)
Aggressive D1 (%)	68 (22.66)	151 (29.72)	15,599 (19.08)
Aggressive D2 (%)	17 (5.66)	45 (8.86)	3412 (4.17)
Aggressive D3 (%)	91 (30.33)	182 (35.83)	24,081 (29.45)
Death (%)	22 (7.33)	52 (10.24)	4084 (4.99)

## Discussion

In the present study, we applied a spatial statistical analysis to compare the geographic patterns of elevated ORs of aggressive PCan across 3 definitions of aggressiveness. To our knowledge, this is the first study to compare 3 different definitions of aggressive PCan in a geospatial context. Our findings have important implications, as identifying geographic areas at significantly higher risk of aggressive PCan may help strategic planning in cancer prevention and control.

Our results suggest five primary findings. First, similar to previous studies [6, 14], we found that variations in the definitions resulted in major differences in the study population numbers, whereby relying on the Gleason scores generally resulted in a higher number of cases defined as aggressive. This variation in the study population size also affected the number and size of clusters demonstrating elevated risk for aggressive PCan in each binomial model. However, the patterns were not dissimilar. We also observed that several clusters did overlap across all models because those census tracts appear to have more aggressive PCan cases regardless of the definition. However, it appears that some clusters could have been overestimated in size because of the high number of cases, notably in definition D3, because numerous “aggressive” cases were based solely on the Gleason score, which is a more subjective measure with changing criteria over time that may not reflect the pathological diagnosis (e.g., staging). The Gleason grading system has been updated several times since its proposal in 1966. Most of the changes were based on new observations and interpretations of the findings that often-broadened inclusion criteria, particularly for “higher grade” cancers [22]. Therefore, the number of cases identified by definitions D1 and D3 will be consistently higher than using SEER summary stage-based definition D2.

Second, we compared statistically significant geographic clusters from binomial models for each definition of aggressive PCan to statistically significant geographic clusters identified in Cox regression models for PCan-specific mortality. Using definition D2 in a binomial model resulted in spatial patterns most similar to results from PCan-specific survival analysis; it also had the highest specificity (98%) with PCan-attributed deaths. D2 also overlapped with clusters from other definitions, presenting with a smaller cluster, an area to potentially target for intervention. This may suggest that definition D2 is not only accurate for detecting the areas with a high disease burden, where patients are more likely to die of PCan (based on spatial analysis), but also could be useful for identifying which areas to target for cancer prevention and control efforts in a limited resource setting. Reasons for this geographic variation in aggressive PCan diagnosis and mortality must be further investigated, under consideration of socioeconomic and environmental area characteristics as well as lifestyle and screening behaviors.

Third, when analyzing the number of deaths in each definition at the patient level and in each cluster, there were more patients with aggressive PCan when using definitions D1 and D3. Thus, these 2 definitions of aggressive PCan had higher sensitivity (D3-79.5; D2-77.2%, respectively) with PCan deaths at the patient level. This finding aligns with the study conducted by Hurwitz and colleagues at the patient level, which compared several definitions based on the number of deaths events, and derived D1 as the most sensitive [6]. However, the geographic clusters from the survival models are more similar to those from binomial models when using definition D2. Therefore, the high number of deaths seen in D1 is likely a result only from the high overall number of cases identified as aggressive by this definition. These definitions (D1 and D3) appear useful on the patient level; but D2 may be more relevant for intervention planning in geospatial studies that are looking at area-level data.

Fourth, we observed complications in definitions of aggressive PCan when using AJCC categories. While Hurwitz et al. argue that AJCC’s TNM categorization is commonly used [6], this system has several issues related to the availability and completness of records in registry data. In contrast to the SEER summary stage (5% missing staging records), far more have been excluded from the analysis when using AJCC’s definition due to missing data (12%). Using Gleason would result in the exclusion of 6% of all cases. Therefore, we suggest that using D2 in when conducting geographical analysis may be preferred because of fewer exclusions.

Finally, there were consistent differences in race/ethnic breakdowns in identified clusters across each definition. The Philadelphia cluster had a much higher percentage of Black patients, for example. This suggests that racial disparities could be playing a role in geographic variation across all 3 definitions. In an additional exploratory analysis, we did adjust clusters by race for each definition, and findings were similar, with the Philadelphia cluster remaining, but shrinking in size (data not shown). Prior studies suggest that the 5 domains of social determinants of health, namely education, economic stability (e.g. living in poverty), access to care (e.g. insurance), social context (discrimination), and neighborhood environment (e.g. toxic exposure, exposures to crime, green space, etc.) could help to explain racial and geographic disparities [23]. This investigation, however, focused first on comparing different definitions of aggressive PCan and how they could change geographic clusters. Future studies are planned that would address the potential contributing factors to each of these clusters in subsequent analyses.

There are several study limitations. First, we reduced the study population by approximately 15% because we needed to exclude all cases where at least one of the three classifications (SEER summary stage, TNM, or Gleason score was missing. Not excluding these cases may result in different geographic patterns, but it would also limit a direct comparison of the definitions. Future studies could explore imputation techniques to aid with missing staging data. Another limitation is that the number of cases was further reduced in the survival analysis because of missing survival information or negative survival times. However, considering the relatively low number of additional exclusions (n = 3,549, 4.2%), we would not expect significant differences in the geographic patterns. Additionally, while we evaluated 3 main definitions of PCan according to the latest recommendations [6], there are other definitions of aggressive PCan that exist that we were not able to incorporate because of their reliance on variables with limited availability in the Pennsylvania cancer registry, specifically the measurement of prostate specific antigen or PSA. While this limits the scope of our investigation, recent literature does suggest that models that do not utilize PSA may outperform those with PSA [6]. Further, in the survival study, patients were followed only until the end of 2017, resulting in a relatively brief follow-up period for several patients (mean 22.7 months). This could affect the sensitivity/specificity of each of our 3 definitions with PCan deaths, given men might not have been followed long enough to die of PCan, particularly for the D1 and D3 definitions. The 5 year-survival rate for prostate cancer is relatively high (~ 98%) for local and regional stage at diagnosis (captured in D1 and D3), but lower for those diagnosed with distant stage (our D2) definition (~ 32%) [24]. A sensitivity analysis we conducted was consistent with literature in that very few, only 2.9% of patients initially diagnosed as aggressive as defined by D2, were still alive after 5 years, compared to 21.6% and 32.3% as defined by D1 and D3, respectively. While this finding further supports the correlation of D2 with risk of death for prostate cancer, future studies with longer follow-up time are needed to evaluate an association between PSA and fatal PCan cases. Additionally, this study was conducted in Pennsylvania only, and results may not be reflected in other states. For example, Pennsylvania has a relatively small population of non-White racial/ethnic groups. Also, in Pennsylvania, members of non-White racial/ethnic groups (including Blacks) primarily reside in the largest urban centers of Philadelphia and Pittsburgh, which may have influenced the location of the identified geographic clusters. Thus, we plan to expand this methodologic investigation in subsequent studies that can explore the impact of race/ethnicity and other social determinants of health on geographic variation in PCan outcomes.

## Conclusion

The definition of aggressive PCan is not universal, and epidemiologists and clinicians may use various criteria and classification schemes. Our findings suggest that differences in the definitions may influence spatial patterns and impact which areas are identified as having an aggressive PCan burden. Even though spatial patterns were not dissimilar across the three definitions, survival analysis showed that geographic clusters of elevated risk of death from PCan were more similar to those found in the model when using the definition D2 based on a SEER summary distant stage. Also, the high degree of overlap between the geographic clusters when using D2 and other definitions in the binomial model suggests that D2 may be a good predictor for aggressive PCan burden and early PCan-specific death. Another advantage of using this definition is that resulted in fewer areas to target for future interventions, which is important given the often-limited resources available for prevention efforts. Finally, we noticed a relatively low number of missing staging information in SEER summary stage definition.

Using a consistent definition will allow for consistent comparisons in future studies. Understanding the impact of differing definitions is important to help address the disparities attributed to aggressive PCan and may impact the planning of public health interventions.

### Electronic supplementary material

Below is the link to the electronic supplementary material.

Supplementary Material 1

## Data Availability

The data that support the findings of this study were derived from patient level, geocoded data from the Pennsylvania State Cancer Registry. Data and code can be made available by contacting Dr. Daniel Wiese (daniel.wiese@cancer.org) and working with the Pennsylvania State Cancer Registry under their current data use agreements, which includes an IRB approval process (ra-dhirb@pa.gov).

## List of abbreviations

CI: 95% Confidence Interval
D1: Definition 1
D2: Definition 2
D3: Definition 3
HR: Hazard Ratio
OR: Odds Ratio
PCan: Prostate Cancer
PCR: Pennsylvania State Cancer Registry
