# Supplementary material for: Defining aggressive prostate cancer: a geospatial perspective
Source: BMC Cancer. 2023 Aug 14;23:754. doi: 10.1186/s12885-023-11281-8 (PMC10424402; doi:10.1186/s12885-023-11281-8)
Supplement: Supplementary file 1 — Supplementary Material 1 [file 12885_2023_11281_MOESM1_ESM.docx]

**Supplementary Materials**

Supplementary Table 1: Comparison between aggressive and non-aggressive PC cases defined by D1.


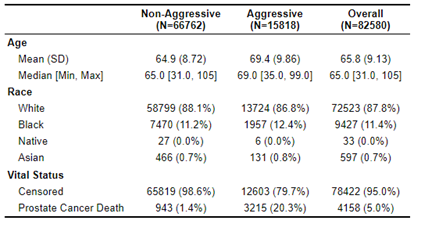


Supplementary Table 2: Comparison between aggressive and non-aggressive PC cases defined by D2.


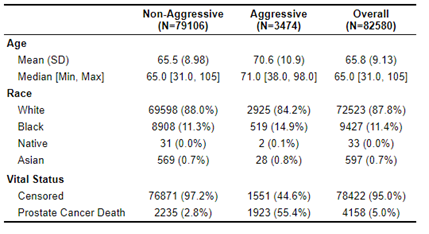


Supplementary Table 3: Comparison between aggressive and non-aggressive PC cases defined by D3.


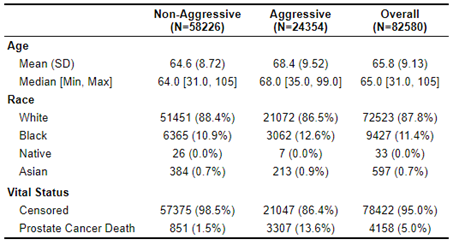


*Supplementary Table 4: Sensitivity/Specificity assessments of the study population for each of the 3 definitions for aggressive prostate cancer and death by prostate cancer.*

|  | **Dead** | **Alive** | **Total** |  |
| --- | --- | --- | --- | --- |
| **D1 Aggressive** | 3215 | 12603 | 15818 |  |
| **D1 not-Aggressive** | 943 | 65819 | 66762 |  |
| **Total** | 4158 | 78422 |  | 82580 |
| **Sensitivity** | 77.3 |  |  |  |
| **Specificity** | 83.9 |  |  |  |
|  | | | | |
|  | **Dead** | **Alive** | **Total** |  |
| **D2 Aggressive** | 1923 | 1551 | 3474 |  |
| **D2 not-Aggressive** | 2235 | 76871 | 79106 |  |
| **Total** | 4158 | 78422 |  | 82580 |
| **Sensitivity** | 46.2 |  |  |  |
| **Specificity** | 98 |  |  |  |
|  | | | | |
|  | **Dead** | **Alive** | **Total** |  |
| **D3 Aggressive** | 3307 | 21047 | 24354 |  |
| **D3 not-Aggressive** | 851 | 57375 | 58226 |  |
| **Total** | 4158 | 78422 |  | 82580 |
| **Sensitivity** | 79.5 |  |  |  |
| **Specificity** | 73.2 |  |  |  |
